# Psychological and Sociodemographic Variables Associated with Increased Anxiety and Anxiety Symptoms in Older Adults: A Scoping Review

**DOI:** 10.3390/geriatrics10040083

**Published:** 2025-06-23

**Authors:** Jesús Enrique Sotelo-Ojeda, Christian Oswaldo Acosta-Quiroz, Raquel García-Flores, Ana Luisa Mónica González-Celis Rangel, Erick Alberto Medina-Jiménez

**Affiliations:** 1 Faculty of Psychology, National Autonomous University of Mexico (UNAM), Mexico City 04510, Mexico; jesussotelo711@gmail.com (J.E.S.-O.); erick_alberto_medina@psicologia.unam.mx (E.A.M.-J.); 2 Department of Psychology, Technological Institute of Sonora (ITSON), Ciudad Obregón 85000, Mexico; raquel.garcia@itson.edu.mx; 3 Head of Psychology—Research and Graduate Division—Psychology of Aging, Quality of Life and Health, FES Iztacala, National Autonomous University of Mexico (UNAM), Mexico City 04510, Mexico; algcr10@gmail.com

**Keywords:** anxiety, older adults, risk factors, protective factors

## Abstract

**Background/Objectives:** There is a high prevalence of anxiety and anxiety symptoms in older adults, which can have cognitive, emotional, and physical repercussions on older adults. It is important to understand the risk factors from psychological variables and sociodemographic variables that may be influencing anxiety symptoms to generate more effective interventions based on modifiable variables. In this context, the objective of this review was to identify psychological and sociodemographic variables as risk factors for anxiety and anxiety symptoms in older adults. **Methods:** The Scoping review followed the guidelines of the (PRISMA-ScR 2018). Five databases were used to reduce bias and identify relevant evidence: Medline via Ovid, PUBMED, CINAHL, PsycINFO, and Web of Science. **Results:** A total of 2150 articles were identified across the five databases; 16 articles were included for data synthesis and methodological quality assessment. **Conclusions:** The variables that maintain the strongest association as both risk and protective factors are age, female sex, physical activity, physical health or medical conditions, depression, perceived and family support, and social and family participation. However, methodological limitations—including inconsistent definitions, diverse and often inadequate measurement tools, and lack of causal inference—restrict the generalizability of findings. These results underscore the need for validated age-appropriate instruments and more rigorous research designs in geriatric anxiety studies.

## 1. Introduction

The tripartite model conceptualizes anxiety and depression symptoms as consisting of three components: (1) general negative affect (i.e., a shared predisposition to experience unpleasant emotions such as fear, sadness, or anger); (2) physiological hyperarousal, which is characteristic of anxiety (e.g., muscle tension, restlessness, or feelings of panic); and (3) reduced positive affect, which is more specific to depression (e.g., decreased interest, energy, and enjoyment of activities) [1,2]. The anxiety is defined as an anticipatory response to a real or imagined threat and is accompanied by muscle tension, vigilance, and cautious behaviors regarding the future [3].

Although the prevalence is usually lower compared to other age groups [4,5], this does not necessarily mean that the risk of developing anxiety (incidence) is also low. The incidence rate of both anxiety and anxiety symptoms in older adult ranges between 3000 and 4000 cases per 100,000 inhabitants worldwide [6]. A meta-analysis of 35 studies conducted in Europe, America, Asia, Africa, and Australia found an anxiety symptom prevalence of 44.6% [7]. The young-old group has a higher prevalence of anxiety cases and symptoms, with just over 4000 cases per 100,000 inhabitants. In the middle-aged group, prevalence is slightly lower, at just under 4000 cases per 100,000 inhabitants, while in the oldest-old group, it may decrease to 3000 cases per 100,000 inhabitants [6].

With the exponential increase in both the incidence of anxiety and the onset of anxiety symptoms across different age groups of older adults, it is essential to consider the various risk factors that may contribute to this increase. Risk factors refer to exposures or characteristics associated with a higher likelihood of a particular outcome and can be controllable or uncontrollable [8]. Among these factors, psychological variables play a prominent role. For example, the relationship between anxiety and quality of life has been shown to be bidirectional, especially in the post-COVID-19 context. A higher quality of life appears to buffer the increase in anxiety, while a high level of anxiety may, in turn, decrease an older adult’s quality of life [9,10,11]. Similarly, depression has been identified as another psychological variable frequently associated with anxiety in older adults, often manifesting in a comorbid manner. Some studies even suggest that anxiety may precede the onset of depression [12,13]. Cognitive impairment has also been bidirectionally linked with anxiety, indicating a complex interaction between these conditions [9,14].

Beyond psychological factors, various social and contextual variables, such as social isolation, loneliness, family relationships, family support and care, communication with neighbors, and bereavement experiences, may also be associated with anxiety in later life; however, the precise nature of these associations remains to be fully established [13,15,16,17,18,19,20]. Furthermore, sociodemographic factors, such as gender, age, place of residence, and educational level, may further influence anxiety levels in older adults [6,15,16,17].

It is important to highlight the lack of systematic reviews or scoping reviews that determine with correct precision which factors within (1) psychological variables and (2) sociodemographic variables have a greater association in older adults and from that knowledge generate optimal interventions for anxiety levels and/or anxiety symptoms. What are the psychological or sociodemographic variables that are a risk factor in the prevalence and increase in anxiety and anxiety symptoms? The objective of this scoping review is to identify psychological and sociodemographic variables as a risk factor in anxiety and anxiety symptoms in older adults.

## 2. Materials and Methods

The scoping review followed PRISMA Extension for Scoping Review (PRISMA-ScR) [21]. The Open Science Framework (OSF) registration is https://osf.io/uqmjv.

### 2.1. Eligibility Criteria

(1) Participants: Studies focusing on populations aged ≥60 years in both men and women were selected.
(2) Exposure/context: Research investigating sociodemographic variables and psychological factors preceding and/or increasing anxiety and anxiety symptoms were included. However, studies on generalized anxiety disorder in the elderly and studies referring to the consequences of anxiety symptoms and/or anxiety in the elderly were excluded. The context considered was the community, including urban and rural areas, retirement homes, nursing homes, and temporary or permanent residences. Studies conducted in hospital settings were not considered.
(3) Study design: Longitudinal and cross-sectional studies written in English were included, without restrictions on the year of publication.
(4) Outcome measures: Studies were eligible if they reported associations between sociodemographic variables and psychological factors, whether these associations were the main focus of the study (primary outcomes) or examined as additional findings (secondary outcomes).
(5) Inclusion criteria.
  (1) Individuals aged over 60 years.
  (2) Studies describing causes or risk factors of anxiety and/or anxiety symptoms.
  (3) Longitudinal and cross-sectional studies.
  (4) Studies conducted in community settings (rural/urban), nursing homes, and temporary residences.
  (5) Studies written in English.

### 2.2. Exclusion Criteria

(1) Hospital setting (studies focused on evaluating anxiety in hospitalized older adults).
(2) Studies where older adults had the following conditions (i.e., cancer, stroke, heart attacks, Parkinson’s, dementia, Alzheimer’s).
(3) Inability to access the full manuscript.

### 2.3. Information Sources

Five databases were used to reduce bias and identify relevant evidence: Medline via Ovid, PUBMED, CINAHL, PsycINFO, and Web of Science. The search was conducted between May and June 2024.

### 2.4. Search

The following MeSH terms were used: exp older adulthood, exp anxiety, exp causality, exp communities (PsycInfo via Ovid). Exp aged, exp anxiety, exp causality, exp independent living (Medline via Ovid). MH aged, MH anxiety, MH risk factors, MH communities (CINAHL EBSCO). MH aged, MH anxiety, MH causality, MH independent living (PUBMED via Ovid). TS aged, TS anxiety, TS causality, TS communities (Web of Science). The complete strategy can be found in File S1.

### 2.5. Selection of Sources of Evidence

All identified citations were collected and uploaded into Rayyan. Title and abstract screening were independently conducted by two researchers, (JS) and (EM), considering inclusion and exclusion criteria. Disagreements were resolved through discussion with a third researcher (CA). Selected articles were then read in full for data synthesis.

### 2.6. Data Charting Process

Data collection was performed independently by two researchers, and agreements were reached in case of discrepancies with a study. Data were collected in a database created ad hoc in Microsoft Excel. Recommendations [22] were considered, focusing on authors/year/country, study design, participants, sociodemographic, and psychological variables, anxiety or anxiety symptoms considered, instruments used, type of data analysis, and results.

### 2.7. Data Items

Following the data collection process, data elements included authors/year/country, study design, participants, types of sociodemographic and psychological variables, anxiety or anxiety symptoms considered, instruments used, type of data analysis, and results.

### 2.8. Critical Appraisal of Individual Sources of Evidence

Methodological quality assessment was carried out using the Critical Appraisal Checklist for Analytical Cross-Sectional Studies and Critical Appraisal Checklist for Cohort Studies [23]. The aim of methodological quality assessment is to identify potential biases in study design, conduct, and analysis. This assessment was conducted by two researchers.

### 2.9. Synthesis of Results

A summary was carried out in table format where the information for each point mentioned in data items was compiled.

## 3. Results

### 3.1. Selection of Sources of Evidence

**Figure 1:**
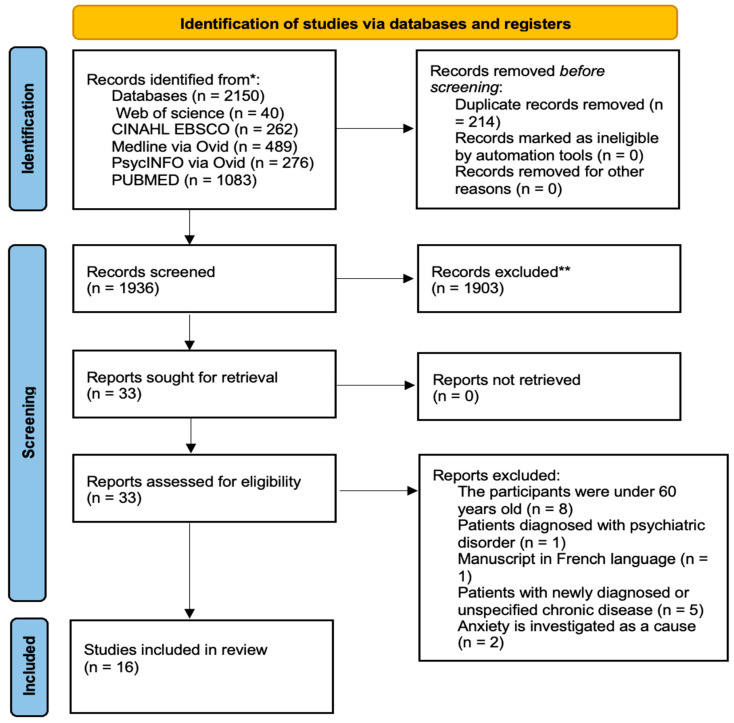
PRISMA flowchart studies selection. * Identified articles, ** Excluded articles.

A total of 2150 articles were identified in the five databases. After excluding 214 duplicate articles, title and abstract screening of 1936 articles resulted in the exclusion of 1903 articles that did not meet the inclusion criteria and were aligned with the exclusion criteria (i.e., individuals under 60 years old, hospital setting, lack of relevance to the systematic review topic). Thirty-three articles were read in full, of which 17 were excluded for reasons such as being in French, involving individuals under 60 years old, focusing on psychiatric diagnoses, not describing chronic diseases, or investigating anxiety as a cause rather than a consequence. A total of 16 articles were included for data synthesis and risk of bias assessment. Figure 1 shows the studies selection flowchart.

### 3.2. Characteristics of Sources of Evidence

The general characteristics of the included studies (N = 16) are presented in Table 1 displays authors/year/country, study design, participants, sociodemographic variables, psychological variables, anxiety or anxiety symptoms, presents authors/year/country, instruments used, and results. Sixteen studies were identified, conducted in Australia, the USA, Poland, Myanmar, Korea, China, Hong Kong, Brazil, Britain, Slovenia, Jordan, and Italy. Notably, no studies were found in Latin America and Central America, highlighting the importance of conducting such research in these regions.

Regarding the years of publication, a good range can be observed, as it spans from the years 1982 to 2024 [24,25]. In this sense, it can be considered a plus for the review since it was not limited by years of search to expand the search strategy. As for the study designs, cross-sectional studies [25,26,27,28,29,30,31], descriptive studies [32], prospective community-based studies [33], and studies not specified in the manuscript [24,34,35,36,37,38,39] were found.

**Table 1:** Characteristics of sources of evidence.

Authors/Year/Country	Research Design	Participants (Age Group) Sample Size	Sociodemographic Variables	Psychological Variables	Anxiety or Anxiety Symptoms	Instruments Used	Outcome
Allcock et al. [25] Australia	A cross-sectional study	N = 303 Total = 70.4 ± 6.2 Male = 72 ± 6.9 Female = 69.67 ± 5.8	The Mediterranean diet (MedDiet)	None	Anxiety symptoms	The DASS-21	We also observed an inverse relationship between legume intake and the severity of anxiety symptoms.
Creighton et al. [28] Australia	A cross-sectional, observational design.	N = 178 Total = 85.4 ± 7.4 Range = 66–101	Age, sex, educational level, and marital status	Perceived Social Support, Social Engagement, Attachment Style, Mastery, Depression, Experience of Negative Life Events, and Experience of a Recent Fall	Anxiety symptoms	Geriatric Anxiety Inventory	The variables with the highest association with anxiety symptoms were generally not modifiable (e.g., attachment style, cognitive impairment).
Cho et al. [29] Myanmar	A cross-sectional study	N = 655 Male = 221 Female = 434	Age, gender, marital status, education level, employment status, social participation, number of friends/relatives met per month, body mass index, vision, dental health, and comorbidity.	Depression	Anxiety symptoms	Geriatric Anxiety Inventory	Association between employment status and anxiety or depression was reported in this study. Elderly participants with poor dental health were at risk for anxiety.
Cybulski et al. [34] Polonia	Not specified in the manuscript	N = 300 Male = 213 (71%) Female = 87 (29%)	Gender, group affiliation, age, and family situation.	Self-efficacy, loneliness, isolation, mourning.	State and Trait anxiety	State-Trait Anxiety Inventory (STAI)	Higher scores in the subscale of anxiety understood as a trait may suggest that the examined were exposed to chronic stressful situations caused.
Kang et al. [33] Korea	Prospective community-based study	Prevalence analysis n = (1204) Incidence analysis n = (566) Persistence analysis n = (343)	Age, gender, living area, and marital status, years of education, housing status, past occupation, current occupation, monthly income, stressful life events, number of chronic medical illnesses, physical inactivity, and drinking problem.	Depression, insomnia, cognitive function, and social support.	Anxiety symptoms	The community version of the Geriatric Mental State Schedule (GMS-B3)	Anxiety symptoms were independently associated with female gender, rented housing, greater number of stressful life events and medical illnesses, physical inactivity, depression, insomnia, and lower cognitive function.
Lu et al. [31] China	A cross-sectional study	N = 1173 individuals Male = (53.6%) Female = (46.4%)	Age, gender, body mass index, educational level, marital status, number of children, pre-retirement occupation, monthly personal income, religion, smoking, physical activity level, physical pain rating, and comorbidities.	Social support, Subjective support, Objective support, Support utilization	Anxiety symptoms	Generalized Anxiety Disorder scale GAD-7	Anxiety was negatively correlated with age, subjective support, support utilization. Female gender showed a higher risk factor for anxiety. Being unemployed before retirement age was a risk factor for anxiety. For social support, we found support utilization to be a protective factor for anxiety and depression.
Cassidy et al. [27] Australia	Cross-sectional study	N = 278 Female = (100%) Range = 70–92	Physical activity, smoking, alcohol consumption, body mass index	Depression	Anxiety symptoms	Beck Anxiety Inventory (BAI)	This study shows that even in later life, a greater level of physical activity is associated with better mood, reduced anxiety and better quality of life.
Colenda y Smith [35] USA	Not specified in the manuscript	N = 123 Male = 54 Female = 69	Age, educational, level total medical comorbidity, and a measure of stressful life events.	Depression, quality of social support, general health status, benzodiazepine use.	State and Trait anxiety	State-Trait Anxiety Inventory (STAI)	Situational factors such as stressful life events, medical comorbidity, and age contributed to higher State Anxiety levels.
Leung et al. [30] Hong Kong	Cross-sectional study	N = 266 >60	Sense of coherence, digital health literacy, information satisfaction, and financial satisfaction, gender, education level, country.	None	Anxiety about the future	Dark Future Scale	The final model in which both DHL were negatively associated with anxiety about the future, while financial satisfaction and information satisfaction had no significant association with anxiety.
Mullins y Lopez [24] USA	Not specified in the manuscript	N = 228 Male = 40.5% Female = 59.5%	Age, education, gender, subjective health, functional ability, length of stay.	Lack of social support	Death Anxiety	Death Anxiety Scale (DAS)	Statistically comparing these proportions, it is clear that older residents are significantly more likely to have high death anxiety than are the younger residents. Interestingly, lack of social support is also associated with higher death anxiety but not in the direction predicted.
Richardson et al. [37] USA	Not specified in the manuscript	N = 377 Male = 258 Female = 119	Age, race, gender, household income, education, marital status, and living arrangement were assessed, physical health and disability, stressful life events, alcohol abuse.	Social support, cognitive impairment, major depressive episode	Anxiety symptoms	The Goldberg Anxiety Scale (GS-A)	However, current MDE was highly associated with anxiety of anxious participants suffered from major depression and only 16% of non-anxious clients had a current MDE.
Da Silva et al. [26] Brazil	Cross-sectional study	N = 200 Male = 156 Female = 44 >60	Physical activity	None	Anxiety symptoms	Hospital Anxiety and Depression Scale (HADS).	This study found that physically active elderly individuals had significantly higher overall QOL scores than their sedentary counterparts, who had the lowest results and a statistically significant relationship with anxiety and depression.
Walters et al. [38] Britain	Not specified in the manuscript	N = 13,349 Male = 39% Female = 61%	Gender, financial stress, functional ability, physical health, housing status, cognitive function, marital status, living alone, high alcohol intake.	None	Anxiety symptoms	General Health Questionnaire (GHQ-28).	Anxiety was significantly associated with female gender, financial stress, functional ability, physical health, lack of confiding relationship, access to help, and negative life events but not age, housing status, cognitive function, marital status, living alone, or high alcohol intake.
Žalik y Zalar [39] Slovenia	Not specified in the manuscript	N = 103 Female = 100%	Living area, elderly clubs, elderly day care centers, elderly homes.	Cognitive status	Anxiety symptoms	Zung self-rating anxiety scale inventory (ASI)	Comparison of the intensity of Zung ASI anxiety symptoms between all the three study groups again showed a statistically significant difference.
Rababa et al. [32] Jordan	Descriptive study	N = 248 Mean age = 63.95 Male = 143 Female = 105	Marital status, gender	Religious coping, spiritual well-being	Death anxiety	Arabic Scale of Death Anxiety (ASDA)	In comparison to male older adults, female older adults reported higher levels of religious coping and lower levels of death anxiety.
Pascut et al. [36] Italy	Not specified in the manuscript	N = 282 Male = 119 Female = 163	Age, sex, nationality, level of education, marital status, job, and the number of people with whom they were living.	Quality-of-life, spirituality well-being, loneliness, fear	Anxiety symptoms	Hospital Anxiety and Depression Scale (HADS)	Anxiety levels were predicted by interrupted or diminished meetings with family/friends during the pandemic. Importance of social support for elderly for the mitigation of their anxiety levels.

### 3.3. Critical Appraisal Within Sources of Evidence

The methodological assessment for cross-sectional studies is presented in Table 2. The studies considered [25,26,27,28,29,30,31,32] generally exhibited good methodological quality as they met the criteria of the Critical Appraisal Checklist for Analytical Cross-Sectional Studies. However, the study by [30] showed four unclear points in certain areas. Additionally, a longitudinal study [33] was evaluated using the Critical Appraisal Checklist for Cohort Studies, as shown in Table 3. The remaining studies [24,34,35,36,37,38,39] did not explicitly define the evaluation design used in the manuscript. However, they were not excluded, as this was not a criterion for exclusion.

### 3.4. Results of Individual Sources of Evidence

#### 3.4.1. Sociodemographic Variables

Age, gender, educational level, marital status, and place of residence (e.g., nursing home, temporary and/or permanent residence, day care centers) have been the most addressed variables [24,28,29,30,31,32,33,34,35,36,37,38,39]. Other related sociodemographic variables addressed as a risk factor for anxiety include dietary habits [25], body mass index (BMI) [27,29,31], socioeconomic status, and occupation [30,33,36,38], dental and vision health problems, and/or controlled disease comorbidities [24,29,31,35,37], physical activity [26,27]. It is worth noting the large number of sociodemographic variables that can influence the increase in anxiety or anxiety symptoms.

#### 3.4.2. Psychological Variables

Psychological variables studied as risk factors for anxiety include depression [27,28,29,35], loneliness and isolation [34,36], perceived support, social support, self-efficacy, spiritual well-being, and quality of life [24,28,31,32,34,35,36,37]. The collected studies show how research focuses on depression, different types of support in older adults, quality of life, and spiritual well-being.

#### 3.4.3. Anxiety or Anxiety Symptoms

An important aspect was to know where the research is oriented regarding anxiety and anxiety symptoms. In this sense, the research shows a greater tendency to investigate anxiety symptoms [25,26,27,28,29,31,33,36,37,38,39], and some studies have focused on anxiety as a state or trait [34,35] as well as anxiety about death [24,32] and anxiety about the future [30].

### 3.5. Synthesis of Results

In Table 4, the main results of sociodemographic variables associated with anxiety and anxiety symptoms can be observed. Age stands out as one of the variables with more studies and influence on anxiety; however, in the analysis of results, there is no segmentation by age groups to determine which age group maintains a higher prevalence of anxiety. Regarding gender, there is a greater influence in females compared to males. Physical activity can be considered another variable with influence on anxiety. As for psychological variables, depression and different types of support for the elderly stand out (Table 5).

## 4. Discussion

The aim of this scoping review was to identify psychological and sociodemographic variables that serve as risk factors for anxiety and anxiety symptoms in older adults. The first aspect to consider is the concept of anxiety and anxiety symptoms, as it aligns with definitions found in the literature [1,2,3,40]. These sources highlight that anxiety and anxiety symptoms can be defined in various ways. In this regard, authors such as [34,35] approach anxiety from the perspective of state-trait anxiety, while others, such as [24,32], focus on anxiety in relation to death. However, the term anxiety symptoms are the most frequently used across studies [24,25,26,27,28,31,33,36,37,38,39], often without a clear conceptual distinction between anxiety, state-trait anxiety, death anxiety, and anxiety symptoms, despite their theoretical differences. This lack of differentiation may hinder a more precise understanding of the specific aspects of anxiety being examined in the research. Another aspect to consider is the wide variety of instruments used (see Table 2) to assess anxiety symptoms in older adults. However, it is crucial to determine whether each instrument effectively measures dimensions of anxiety symptoms [1,2].

Previous studies on quality of life [9,10] align with findings from the scoping review [26,36], indicating that higher quality of life is associated with lower anxiety symptoms. Depression, which has often been studied alongside anxiety, was previously considered a comorbid condition in which anxiety predicted depressive symptoms [1,2,13]. However, in this review, depression was found to be a risk factor for anxiety symptoms [27,33,35,37] and this bidirectional model is supported by the meta-analytic findings [41], who showed that anxiety and depression are mutually reinforcing risk factors over time, suggesting a dynamic and reciprocal interaction that warrants further exploration. A key finding was the protective role of different types of social support, including perceived support, family support, and social participation, in reducing anxiety symptoms [24,29,31,34,35,36,38].

Age was the most frequently studied sociodemographic variable. Some studies [24,31,34,35,37] found a relationship between anxiety and age. However, only one study [33] reported higher levels of anxiety symptoms with increasing age. This finding contradicts previous research suggesting that anxiety tends to decrease with age [6]. Therefore, future studies should emphasize identifying the age group with the highest prevalence of anxiety symptoms to avoid generalizing prevalence and incidence across the entire older adult population. Regarding gender, studies [31,32,33,34,38] report a higher prevalence of anxiety symptoms in women compared to men. This discrepancy may be due to greater vulnerability in women related to higher negative affectivity, emotional expression during upbringing, and a stronger tendency toward worry and rumination [42], added to this is the presence of moderating factors such as recent stressful life events (e.g., bereavement, retirement, loss of autonomy) [43], and social factors such as loneliness or lack of social support [44]. These elements can contribute to maintaining or even increasing anxiety symptoms in older adults [45]. Physical activity [26,31,33,36] was identified as a protective factor, whereas physical health conditions [24,29,31,33,35,37] were considered risk factors. That is, chronic illnesses were associated with higher anxiety symptoms. Other variables, such as age, education level, and place of residence, require further investigation to determine their influence on anxiety symptoms.

Notably, depression and loneliness have well-defined protective and risk factors. Protective factors include physical activity and social support, but more research is needed to identify additional protective factors against depression and loneliness. In contrast, established risk factors include social isolation, chronic illnesses, bereavement, low socioeconomic status, female gender, and family violence [46,47,48,49].

### Limitations

One limitation was the lack of bias assessment in the reviewed studies [17,27,28,29,30,31,32], as they did not explicitly describe their methodology. Another limitation was the inability to establish causal relationships between sociodemographic and psychological variables and anxiety symptoms due to the nature of the reviewed studies. While associations were identified, causality could not be determined. Additionally, the assessment of anxiety in older adults presents several challenges. Many commonly used instruments were originally developed for younger populations and may not account for age-related differences, such as a higher prevalence of somatic symptoms, comorbid physical conditions, and the frequent overlap between anxiety and depression [50]. Most tools lack adequate discriminant validity, test–retest reliability, and age-specific normative data, which limits their diagnostic utility in geriatric populations. Measures like the BAI and PSWQ show some promise, while instruments such as the GMSE, GAI, and WS—specifically developed for older adults—demonstrate stronger psychometric properties, though further validation is still needed [51]. These limitations underscore the urgent need for anxiety measures that are both psychometrically robust and appropriate for older adults. Furthermore, the reviewed studies did not specify the types of variables that increase or decrease as protective or risk factors before and during the COVID-19 pandemic, despite evidence indicating that this period has a differential influence on these variables [52].

## 5. Conclusions

- Anxiety symptoms are the most frequently studied aspect of anxiety in older adults, yet a wide range of assessment tools is used.
- Prevalence and incidence of anxiety symptoms in older adults have been generalized across the population. However, only one study identified a higher prevalence in the oldest-old group.
- The variables most strongly associated with anxiety—either as risk or protective factors—are age, female gender, physical activity, physical health conditions, depression, perceived and family support, and social participation.
- New variables linked to anxiety include body mass index (BMI) and dietary habits.

## Figures and Tables

**Table 2:** Methodological quality in cross-sectional study.

Question	Allcock [25]	Da Silva [26]	Cassidy [20]	Creighton [27]	Leung [30]	Lu [31]	Cho [29]	Rababa [32]
Were the criteria for inclusion in the sample clearly defined?	NOT	YES	YES	YES	UNCLEAR	YES	YES	NOT
Were the study subjects and the setting described in detail?	YES	UNCLEAR	YES	YES	UNCLEAR	YES	YES	YES
Was the exposure measured in a valid and reliable way?	YES	YES	YES	YES	UNCLEAR	YES	UNCLEAR	YES
Were objective, standard criteria used for measurement of the condition?	YES	YES	YES	YES	YES	YES	YES	YES
Were confounding factors identified?	YES	NOT	YES	YES	YES	YES	YES	YES
Were strategies to deal with confounding factors stated?	YES	NOT	YES	YES	UNCLEAR	YES	YES	YES
Were the outcomes measured in a valid and reliable way?	YES	YES	YES	YES	YES	YES	YES	YES
Was appropriate statistical analysis used?	YES	YES	YES	YES	YES	YES	YES	YES

**Table 3:** Methodological quality in cohort study.

Question	Kang [33]
Were the two groups similar and recruited from the same population?	NOT
Were the exposures measured similarly to assign people to both exposed and unexposed groups?	YES
Was the exposure measured in a valid and reliable way?	YES
Were confounding factors identified?	YES
Were strategies to deal with confounding factors stated?	YES
Were the groups/participants free of the outcome at the start of the study (or at the moment of exposure)?	UNCLEAR
Were the outcomes measured in a valid and reliable way?	YES
Was the follow up time reported and sufficient to be long enough for outcomes to occur?	YES
Was the follow up complete, and if not, were the reasons to loss to follow up described and explored?	YES
Were strategies to address incomplete follow up utilized?	UNCLEAR
Was appropriate statistical analysis used?	YES

**Table 4:** Results of sociodemographic variables associated with anxiety and anxiety symptoms.

Sociodemographic Variables	Studies	Is There an Association in Each Study?
Age	Creighton et al. [28] Colenda y Smith [35] * Cybulski et al. [34] * Kang et al. [33] * Cho et al. [29] Lu et al. [31] * Mullins y Lopez [24] * Richardson et al. [37] * Walters et al. [38]	6 (9)
Gender	Creighton et al. [28] Cybulski et al. [34] * Kang et al. [33] * Lu et al. [31] * Walters et al. [38] * Rababa et al. [32] *	5 (6)
Educational level	Creighton et al. [28] Mullins y Lopez [24] * Walters et al. [38] Rababa et al. [32] *	2 (4)
Marital status	Creighton et al. [28] Cho et al. [29] Walters et al. [38]	0 (3)
Place of residence	Creighton et al. [28] * Kang et al. [33] * Walters et al. [38] Zalik y Zalar, [39] *	3 (4)
Body mass index	Cho et al. [29] *	1 (1)
Socioeconomic level/employment	Cho et al. [29] * Leung et al. [30] Lu et al. [31] * Walters et al. [38] *	3 (4)
Food type	Allcock et al. [25] *	1 (1)
Physical activity	Cassidy et al. [27] * Da Silva et al. [26] * Kang et al. [33] * Lu et al. [31] * Pascut et al. [36] *	5 (5)
Physical health/medical condition	Cho et al. [29] * Kang et al. [33] * Lu et al. [31] * Colenda y Smith, [35] * Mullins y Lopez [24] * Richardson et al. [37] * Walters et al. [38] * Rababa et al. [32] *	8 (8)

Note: () is the total number of studies that address a certain variable. * There is an association in the study.

**Table 5:** Results of psychological variables associated with anxiety.

Psychological Variables	Authors	Is There an Association in Each Study?
Depression	Cassidy et al. [27] * Colenda y Smith, [35] * Kang et al. [33] * Richardson et al. [37] *	4 (4)
Loneliness	Cybulski et al. [34] * Pascut et al. [36] *	2 (2)
Isolation	Cybulski et al. [34] *	1 (1)
Types of support/social participation/family (F-P)	Cho et al. [29] * Cybulski et al. [34] * Colenda y Smith, [35] * Lu et al. [31] * Mullins y Lopez [24] * Walters et al. [38] * Pascut et al. [36] *	7 (7)
Quality of life (F-P)	Da Silva et al. [26] * Pascut et al. [36] *	2 (2)
Spiritual well-being (F-P)	Rababa et al. [32] * Pascut et al. [36] *	2(2)
Self-efficacy (F-P)	Cybulski et al. [34] *	1 (1)

Note: () is the total number of studies that address a certain variable. * There is an association in the study. (F-P) is considered a protective factor.

## Supplementary Materials

The following supporting information can be downloaded at: https://www.mdpi.com/article/10.3390/geriatrics10040083/s1. File S1: Availability of data, code, and other materials.

## References

[1] Hollander-Gijsman M.D., De Beurs E., Van Der Wee N., Van Rood Y., Zitman F. (2009). Distinguishing between depression and anxiety: A proposal for an extension of the tripartite model. Eur. Psychiatry.

[2] Clark L.A., Watson D. (1991). Tripartite model of anxiety and depression: Psychometric evidence and taxonomic implications. J. Abnorm. Psychol..

[3] Asociación Americana de Psiquiatría (2014). Manual Diagnóstico y Estadístico de los Trastornos Mentales.

[4] Byrne G.J. (2002). What happens to anxiety disorders in later life?. Braz. J. Psychiatry.

[5] Canuto A., Weber K., Baertschi M., Andreas S., Volkert J., Dehoust M.C., Sehner S., Suling A., Wegscheider K., Ausín B. (2017). Anxiety Disorders in Old Age: Psychiatric Comorbidities, Quality of Life, and Prevalence According to Age, Gender, and Country. Am. J. Geriatr. Psychiatry.

[6] Javaid S.F., Hashim I.J., Hashim M.J., Stip É., Samad A., Ahbabi A.A. (2023). Epidemiology of anxiety disorders: Global burden and sociodemographic associations. Middle East Curr. Psychiatry.

[7] Jalali A., Ziapour A., Karimi Z., Rezaei M., Emami B., Kalhori R.P., Khosravi F., Sameni J.S., Kazeminia M. (2024). Global prevalence of depression, anxiety, and stress in the elderly population: A systematic review and meta-analysis. BMC Geriatr..

[8] Aromataris E., Lockwood C., Porritt K., Pilla B., Jordan Z. (2024). JBI Manual for Evidence Synthesis. JBI. https://synthesismanual.jbi.global.

[9] Grassi L., Caruso R., Da Ronch C., Härter M., Schulz H., Volkert J., Dehoust M.C., Sehner S., Suling A., Wegscheider K. (2020). Quality of life, level of functioning, and its relationship with mental and physical disorders in the elderly: Results from the MentDis_ICF65+ study. Health Qual. Life Outcomes.

[10] Sani F.N., Belo A.M.A., Susanti Y., Ulkhasanah M.E. The relationship of anxiety level with quality of life in elderly. Proceedings of the International Conference on Nursing and Health Sciences.

[11] Siew S.K.H., Mahendran R., Yu J. (2021). Directional Effects of Social Isolation and Quality of Life on Anxiety Levels Among Community-Dwelling Older Adults During a COVID-19 Lockdown. Am. J. Geriatr. Psychiatry.

[12] Byrne G.J. (2017). Anxiety in late life. Ment. Health Illn. Elder..

[13] Chew-Graham C.A. (2016). Anxiety depression in older people: Diagnostic challenges. Ment. Health Older People Guide Prim. Care Pract..

[14] Sun L., Li W., Qiu Q., Hu Y., Yang Z., Xiao S. (2023). Anxiety adds the risk of cognitive progression and is associated with axon/synapse degeneration among cognitively unimpaired older adults. eBioMedicine.

[15] Byeon H. (2021). Exploring Factors for Predicting Anxiety Disorders of the Elderly Living Alone in South Korea Using Interpretable Machine Learning: A Population-Based Study. Int. J. Environ. Res. Public. Health.

[16] Jalil J., Volle D., Zhu T., Sassounian M. (2024). Depression, anxiety, and other mood disorders. Geriatr. Med..

[17] He Z., Tan W., Ma H., Shuai Y., Shan Z., Zhai J., Qiu Y., Zeng H., Chen X., Wang S. (2023). Prevalence and factors associated with depression and anxiety among older adults: A large-scale cross-sectional study in China. J. Affect. Disord..

[18] Paukert A.L., Pettit J.W., Kunik M.E., Wilson N., Novy D.M., Rhoades H.M., Greisinger A.J., Wehmanen O.A., Stanley M.A. (2010). The Roles of Social Support and Self-Efficacy in Physical Health’s Impact on Depressive and Anxiety Symptoms in Older Adults. J. Clin. Psychol. Med. Settings.

[19] Robb C.E., De Jager C.A., Ahmadi-Abhari S., Giannakopoulou P., Udeh-Momoh C., McKeand J., Price G., Car J., Majeed A., Ward H. (2020). Associations of Social Isolation with Anxiety and Depression During the Early COVID-19 Pandemic: A Survey of Older Adults in London, UK. Front. Psychiatry.

[20] Tragantzopoulou P., Giannouli V. (2021). Social isolation and loneliness in old age: Exploring their role in mental and physical health. Psychiatriki.

[21] Tricco A.C., Lillie E., Zarin W., O’Brien K.K., Colquhoun H., Levac D., Moher D., Peters M.D., Horsley T., Weeks L. (2018). PRISMA Extension for Scoping Reviews (PRISMA-ScR): Checklist and Explanation. Ann. Intern. Med..

[22] Arksey H., O’malley L. (2005). Scoping studies: Towards a methodological framework. Int. J. Soc. Res. Methodol..

[23] Moola S., Munn Z., Tufanaru C., Aromataris E., Sears K., Sfetcu R., Currie M., Qureshi R., Mattis P., Lisy K., Aromataris E., Munn Z. (2020). Systematic Reviews of Etiology and Risk. JBI Manual for Evidence Synthesis.

[24] Mullins L.C., Lopez M.A. (1982). Death anxiety among nursing home residents: A comparison of the young-old and the old-old. Death Educ..

[25] Allcock L., Mantzioris E., Villani A. (2024). Adherence to a Mediterranean Diet Is Inversely Associated with Anxiety and Stress but Not Depression: A Cross-Sectional Analysis of Community-Dwelling Older Australians. Nutrients.

[26] de Oliveira L.D., Souza E.C., Rodrigues R.A., Fett C.A., Piva A.B. (2019). The effects of physical activity on anxiety, depression, and quality of life in elderly people living in the community. Trends Psychiatry Psychother..

[27] Cassidy K., Kotynia-English R., Acres J., Flicker L., Lautenschlager N.T., Almeida O.P. (2004). Association between lifestyle factors and mental health measures among community-dwelling older women. Aust. N. Z. J. Psychiatry.

[28] Creighton A.S., Davison T.E., Kissane D.W. (2018). The Factors Associated with Anxiety Symptom Severity in Older Adults Living in Nursing Homes and Other Residential Aged Care Facilities. J. Aging Health.

[29] Cho S.M., Saw Y.M., Saw T.N., Than T.M., Khaing M., Khine A.T., Kariya T., Soe P.P., Oo S., Hamajima N. (2021). Prevalence and risk factors of anxiety and depression among the community-dwelling elderly in Nay Pyi Taw Union Territory, Myanmar. Sci. Rep..

[30] Leung A.Y.M., Parial L.L., Tolabing M.C., Sim T., Mo P., Okan O., Dadaczynski K. (2021). Sense of coherence mediates the relationship between digital health literacy and anxiety about the future in aging population during the COVID-19 pandemic: A path analysis. Aging Ment. Health.

[31] Lu L., Shen H., Tan L., Huang Q., Chen Q., Liang M., He L., Zhou Y. (2023). Prevalence and factors associated with anxiety and depression among community-dwelling older adults in Hunan, China: A cross-sectional study. BMC Psychiatry.

[32] Rababa M., Hayajneh A.A., Bani-Iss W. (2020). Association of Death Anxiety with Spiritual Well-Being and Religious Coping in Older Adults During the COVID-19 Pandemic. J. Relig. Health.

[33] Kang H., Bae K., Kim S., Shin I., Yoon J., Kim J. (2015). Anxiety symptoms in Korean elderly individuals: A two-year longitudinal community study. Int. Psychogeriatr..

[34] Cybulski M., Cybulski L., Krajewska-Kulak E., Cwalina U. (2017). The level of emotion control, anxiety, and self-efficacy in the elderly in Bialystok, Poland. Clin. Interv. Aging.

[35] Colenda C.C., Smith S.L. (1993). Multivariate Modeling of Anxiety and Depression in Community-Dwelling Elderly Persons. Am. J. Geriatr. Psychiatry.

[36] Pascut S., Feruglio S., Crescentini C., Matiz A. (2022). Predictive Factors of Anxiety, Depression, and Health-Related Quality of Life in Community-Dwelling and Institutionalized Elderly during the COVID-19 Pandemic. Int. J. Environ. Res. Public Health.

[37] Richardson T.M., Simning A., He H., Conwell Y. (2010). Anxiety and its correlates among older adults accessing aging services. Int. J. Geriatr. Psychiatry.

[38] Walters K., Breeze E., Wilkinson P., Price G.M., Bulpitt C.J., Fletcher A. (2004). Local Area Deprivation and Urban–Rural Differences in Anxiety and Depression Among People Older Than 75 Years in Britain. Am. J. Public Health.

[39] Zalik E., Zalar B. (2013). Differences in mood between elderly persons living in different residential environments in Slovenia. Psychiatr. Danub..

[40] Spielberger C., Gorsuch R., Lushene R. (1970). STAI: Manual for the State-Trait Anxiety Inventory.

[41] Jacobson N.C., Newman M.G. (2017). Anxiety and depression as bidirectional risk factors for one another: A meta-analysis of longitudinal studies. Psychol. Bull..

[42] Craske M.G. (2003). Why More Women Than Men?. Elsevier eBooks.

[43] Charles S.T., Carstensen L.L. (2009). Social and Emotional Aging. Annu. Rev. Psychol..

[44] Cacioppo J.T., Hawkley L.C. (2009). Perceived social isolation and cognition. Trends Cogn. Sci..

[45] Wetherell J.L., Gatz M., Pedersen N.L. (2001). A longitudinal analysis of anxiety and depressive symptoms. Psychol. Aging.

[46] Abdoli N., Salari N., Darvishi N., Jafarpour S., Solaymani M., Mohammadi M., Shohaimi S. (2021). The global prevalence of major depressive disorder (MDD) among the elderly: A systematic review and meta-analysis. Neurosci. Biobehav. Rev..

[47] Dahlberg L., McKee K.J., Frank A., Naseer M. (2021). A systematic review of longitudinal risk factors for loneliness in older adults. Aging Ment. Health.

[48] George S., Augustine A., Kumar S. (2020). Late-life depression: Epidemiology, assessment and diagnosis. J. Geriatr. Care Res..

[49] Maier A., Riedel-Heller S.G., Pabst A., Luppa M. (2021). Risk factors and protective factors of depression in older people 65+. A systematic review. PLoS ONE.

[50] Balsamo M., Cataldi F., Carlucci L., Fairfield B. (2018). Assessment of anxiety in older adults: A review of self-report measures. Clin. Interv. Aging.

[51] Therrien Z., Hunsley J. (2012). Assessment of anxiety in older adults: A systematic review of commonly used measures. Aging Ment. Health.

[52] Giannoulis K., Giannouli V. (2021). Religiosity and spirituality in the era of the COVID-19 pandemic: An overview of exploring emotional parameters. Encephalos.

