# Supplementary material for: Psychological and Sociodemographic Variables Associated with Increased Anxiety and Anxiety Symptoms in Older Adults: A Scoping Review
Source: Geriatrics (Basel). 2025 Jun 23;10(4):83. doi: 10.3390/geriatrics10040083 (PMC12286065; doi:10.3390/geriatrics10040083)
Supplement: Supplementary file 1 [file geriatrics-10-00083-s001.zip › geriatrics-3597088-supplementary.pdf]

Availability of data, code, and other materials.

Supplementary data 1

APA PsycInfo <1806 to May Week 1 2024>

https://ovidsp.ovid.com/ovidweb.cgi?T=JS&NEWS=N&PAGE=main&SHAREDSEARCHID=2mK99fh8GzzKsIUvdvbziKxBPUpNrLCBzEqHuv8nYxITPBQcVKuHRkQESxIa2Ojau

#	Consulta	Resultados de 2 May 2024
1	exp older adulthood/	18,920
2	Older Adult.mp.	7,602
3	Elder$.mp.	86,880
4	Old*.mp.	607,136
5	Aged+65.mp.	10,614
6	exp anxiety/	95,456
7	anxious.mp.	25,540
8	anxiety symptoms.mp.	14,630
9	Anxie*.mp.	304,045
10	exp causality/	5,803
11	risk factor.mp.	39,315
12	predict*.mp.	559,668
13	correlation.mp.	148,902
14	exp communities/	53,151
15	community-Dwelling.mp.	12,895
16	Community Health Centers.mp.	1,406
17	1 or 2 or 3 or 4 or 5	652,409
18	6 or 7 or 8 or 9	311,145
19	10 or 11 or 12 or 13	716,759
20	14 or 15 or 16	66,345
21	17 and 18 and 19 and 20	276

Ovid MEDLINE(R) <1946 to May Week 1 2024>

https://ovidsp.ovid.com/ovidweb.cgi?T=JS&NEWS=N&PAGE=main&SHAREDSEARCHID=sVEdMkxeBovnVs4jJv1t2q1fRuwdJ3VglyY2UrLGlz7FeeWbXLCckaLFlzk9JFtn

#	Consulta	Resultados de 2 May 2024
1	exp Aged/	3,493,942
2	Elderly.mp.	274,267
3	Older Adult.mp.	10,831
4	Old*.mp.	1,545,000
5	Aged+65.mp.	31,112
6	exp Anxiety/	117,410
7	anxious.mp.	18,341
8	anxiety symptoms.mp.	12,685
9	Anxie*.mp.	281,361
10	exp causality/	999,913
11	risk factor.mp.	242,993
12	predict*.mp.	1,854,985
13	correlation.mp.	917,749
14	exp Independent Living/	12,290
15	community-Dwelling.mp.	29,537
16	Community Health Centers.mp.	8,881
17	Nursing home.mp.	24,573
18	1 or 2 or 3 or 4 or 5	4,579,823
19	6 or 7 or 8 or 9	288,023
20	10 or 11 or 12 or 13	3,496,515
21	14 or 15 or 16 or 17	65,809
22	18 and 19 and 20 and 21	489

CINAHL EBSCO

<1806 to May Week 1 2024>

#	Query	Results
S16	S12 AND S13 AND S14AND S15	262
S15	S9 OR S10 OR S11	121,449
S14	S7 OR S8	245,034
S13	S4 OR S5 OR S6	113,618
S12	S1 OR S2 OR S3	1,083,502
S11	TX Community HealthCenters	32,778
S10	TX community-Dwelling	30,574
S9	(MH "Communities+")	61,228
S8	TX causality	19,368
S7	(MH "Risk Factors+")	226,650
S6	TX anxiety symptoms	38,528
S5	TX anxious	31,668
S4	(MH "Anxiety+")	65,777
S3	TX Older Adult	139,334
S2	TX Elderly	190,584
S1	(MH "Aged+")	956,012

Pubmed via Ovid

<1968 to May Week 1 2024>

Actions	Details	Query	Results	Time
•••	›	Search: ((((((((aged[MeSH Terms]) OR (Elderly[Text Word])) OR (Aged, 80 and over[Text Word])) AND (anxiety[MeSH Terms])) OR (anxiety symptoms[Text Word]))) OR (anxious[Text Word]) AND (causality[MeSH Terms]))) OR (risk factor[Text Word])) OR (correlation[Text Word]) AND (Independent living[MeSH Terms])	1,083	20:31:29

Web Of Science

https://www.webofscience.com/wos/woscc/summary/75b98e6a-efb6-45e2-9a11-9ca62ddee89b-efefc187/relevance/1

Web of Science Search Strategy (v0.1)
Database: Web of Science Core Collection
Entitlements:
- WOS.SCI: 1900 to 2024
- WOS.AHCI: 1975 to 2024
- WOS.BHCI: 2005 to 2024
- WOS.BSCI: 2005 to 2024
- WOS.ESCI: 2019 to 2024
- WOS.ISTP: 1990 to 2024
- WOS.SSCI: 1900 to 2024
- WOS.ISSHP: 1990 to 2024

Searches:

1: TS=(aged OR older adult.mp. OR Elder*.mp. OR old*.mp.) AND TS=(Anxiety OR anxious.mp. OR Anxie*.mp. OR anxiety symptoms.mp.) AND TS=(Causality OR risk factor.mp. OR predict*.mp. OR correlation.mp.) AND TS=(communities OR community-Dwelling.mp. OR Community Health Centers.mp.)

Date Run: Mon Jun 03 2024 10:23:22 GMT-0600 (hora estándar central) Results: 40
